# Meetings of Societies

**Published:** 1937

**Authors:** 


### Meetings of Societies

### Bristol Medico-Chirurgical Society

The fourth meeting of the Session was held on Wednesday, January 13th at 8.30 p.m.

Dr. Richard Clarke was in the chair, and fifty-three members were present.

Dr. Price showed pathological specimens and Dr. Bergin showed Skiagrams.

Mr. Wilfrid Adams read a paper on "**Spinal Anæsthesia,**" to open a discussion in which joined Prof. Rendle Short, Mr. Kindersley, Mr. Ferrier Walters, Dr. Corfield, Mr. Paul, Mr. Eric Watson-Williams, Mr. Leonard Moore and Mr. Cooke, and Mr. Adams replied to many questions.

The fifth meeting of the Session was held on Wednesday, February 10th, at 8.30 p.m.

Dr. Richard Clarke was in the chair, and one hundred and twenty-six members were present.

Dr. Mayes showed Skiagrams, and Professor Davie showed pathological specimens.

The President introduced the Lecturer, Dr. John Parkinson of the London Hospital, who gave his address on "**Cardiac Pain.**"

Dr. Herapath proposed and Dr. Bruce Perry seconded a vote of thanks to Dr. Parkinson for his lecture.

The sixth meeting of the Session was held on Wednesday, March 10th, at 8.30 p.m.

Dr. Richard Clarke was in the chair, and sixty-three members were present.

Dr. Bush showed Skiagrams and Dr. A. L. Taylor showed pathological specimens.

Mr. C. Ferrier Walters read a paper entitled: "**The enlarged prostate from the point of view of Practitioner and Surgeon.**" In a brief discussion which followed Mr. Moore, Mr. Adams and Mr. Foss took part.

Mr. H. K. Pridie then showed a cinematograph film demonstrating the Hey Groves method of introduction of the modified Smith-Peterson pin in the treatment of intra-capsular fracture of the neck of the femur.

